# Editorial: Extracellular vesicles in diseases, host-pathogen interaction and therapeutic applications

**DOI:** 10.3389/fcimb.2022.1010008

**Published:** 2022-09-22

**Authors:** Lysangela R. Alves, Alejandro Correa, Allan J. Guimarães, Marcio L. Rodrigues

**Affiliations:** ^1^ Gene Expression Regulation Laboratory, Instituto Carlos Chagas - FIOCRUZ-PR, Curitiba, Brazil; ^2^ Stem Cells Basic Biology Laboratory, Instituto Carlos Chagas - FIOCRUZ-PR, Curitiba, Brazil; ^3^ Laboratório de Bioquímica e Imunologia das Micoses, Departamento de Microbiologia e Parasitologia, Instituto Biomédico, Universidade Federal Fluminense, Rio de Janeiro, Brazil; ^4^ Instituto de Microbiologia Paulo de Góes (IMPG), Universidade Federal do Rio de Janeiro, Niteroi, Brazil

**Keywords:** extracellular vescicles, infectious Disease, non-infectious diseases, cell communication, therapeutic, biodelivery

Extracellular vesicles (EVs) have emerged as key players in the biology of eukaryotes and prokaryotes. A simple Pubmed (https://pubmed.ncbi.nlm.nih.gov) search using the terms extracellular vesicles, exosomes, or microvesicles suggests a constantly changing field that, in two decades, has jumped from less than 100 scientific publications in 2002 to more than 8,000 in 2021. EVs are the mediators in cell-cell communication and host-pathogen interactions.

Pathogens can use EVs to communicate, which facilitates the persistence of infection, impacts pathogen motility, and determines tissue tropism (Cipriano and Hajduk, 2018). In the intracellular milieu, pathogens can alter the homeostasis of host cells, inducing the differential expression of RNAs and proteins (Rodrigues et al., 2015; Munhoz da Rocha et al., 2020). EVs originating from infected cells can modulate the immune response and also affect host membrane properties (Cipriano and Hajduk, 2018). EVs are also important for non-infectious diseases, including atherosclerosis, rheumatic diseases, diabetes, kidney diseases, and cancer (Marti and Johnson, 2016; Zhang et al., 2016; Cosenza et al., 2017; Rashed et al., 2017; Jiang et al., 2022). As already mentioned, the current interest in EV biology is growing exponentially, and there are ongoing efforts to uncover the potential use of these structures for the biodelivery of small molecules for therapeutic purposes (Kutralam-Muniasamy et al., 2015).

The current Frontiers Research Topic efficiently illustrates the summarized multiplicity of EVs above. Jing et al. comprehensively review the role of EVs during sepsis, a syndrome that is defined as an organ dysfunction with a high risk of death, caused by a dysregulated host response to an infection. EVs in this and other models are vehicles of exportation of proteins, miRNAs, and immunomodulators that present pro- or anti-inflammatory effects in the recipient cells, which promote sepsis-specific changes in the organism. The review also highlights the potential biotechnological application of EVs in the sepsis response.

In addition to reviewing to role of EVs in sepsis, this Frontier’s volume also covers experimental models in the same field. Fitzpatrick et al. study the role of EVs and endothelial cells in sepsis progression. They show that upon *Staphylococcus aureus* infection, endothelial cells-derived exosomes induce a pro-inflammatory response in monocytes, with the consequent expression of CD11b and MHCII, and results in the dysregulation of cytokine secretion during sepsis. In addition, the microRNA-99 (miR-99) is shown to be enriched in EVs in response to *S. aureus* infection. This is associated with a pro-inflammatory phenotype and cytokine release involving the target of rapamycin (mTOR) protein.

Eukaryotic pathogens that produce EVs are also investigated in the current Research Topic. Octaviano et al. study the role of EVs in the human pathogenic fungi *Paracoccidioides brasiliensis* using an avirulent strain (aPb18). After incubating the aPb18 strain with virulent strain-derived EVs (vEVs), the isolated avirulent strain recovers the ability to grow under oxidative and nitrosative stresses. In addition, the aEVs induce the expression of inflammatory mediators in macrophages as well as in mice. Pre-treating mice with aEVs exacerbate the infections, as the number of colonies formed in the lungs are higher. This effect is accompanied by increased concentrations of TNF-α, IFN-γ, IL-6, and MCP-1.

Dantas-Pereira et al. review the impact of EV release by pathogen or host cells on the immune response to parasites. In *Trypanosoma cruzi*, the causative agent of Chagas disease (CD), it is shown that EVs contain parasite antigens. The EVs derived from both parasite and host cells during infection are able to promote *T. cruzi* evasion by inhibiting complement-mediated parasite lysis. The EVs impact not only the physiology of the infected, but also the neighbor cells, through the induction of a proinflammatory phenotype. So far, the available evidence indicates that the parasite EVs can act both activating the host immune response and promoting pathogenesis.

Infection is not the only condition discussed in the set of studies published in this Research Topic. Ding et al. show that EVs are also crucial for genetic and chronic diseases. The Amyloid precursor protein (APP) protein is important for the development of Alzheimer’s disease (AD). EVs derived from AD mouse brain promote APP expression in neuronal cells. The EVs carrying APP induce the dysregulation of genes related to pathogenesis, cell migration and invasion in normal cells by reducing the levels of the miRNA miR-185-5p. Reduced levels of miR-185-p is already observed in the serum of both AD mice and patients, indicating an important role of EVs and their use as potential therapeutic targets and/or biomarkers in the set of Alzheimer’s disease.

Kusuma et al. undertook an analysis of the impact of 2D and 3D mesenchymal stem cells (MSCs) culture on EV cargo and functional properties. A model of lung injury in aged mice treated with 3D EVs shows no improvement in lung function. The lung tissue of these animals shows increased collagen deposition, myofibroblast differentiation and leukocyte infiltration. EV proteomics reveals different content levels of immune-derived and fibrosis/extracellular matrix/membrane organization components between EVs from 2D and 3D cultures, which concurs with the *in vivo* experiments.

Fan et al. review the pathogenesis of cardiac fibrosis and the potential of EVs for diagnosis and treatment. Cardiac fibrosis is a complex condition that is implicated in the deposition of abnormal amounts of extracellular matrix, in addition to the proliferation of cardiac fibroblasts. Growing evidence points to the notion that EVs are important players in cardiac fibrosis. Remarkably, they carry disease-specific proteins and miRNA, suggesting their potential use as biomarkers. Their use as drug delivery systems is also under discussion.

In summary, these studies endorse the functional versatility of EVs in pathophysiological conditions. EVs can be isolated from all body fluids and interact with multiple cell types, which reinforces their potential as disease biomarkers and functional players. In this direction, the role of EVs in tissue regeneration, disease progression or control, and pathogenic processes point to multiple applications in diagnostics, drug delivery, and as vaccine platforms. We invite the readers to navigate through the various and remarkable features of the EV research.

## Author contributions

LA, AG, AC, MR wrote, corrected and aprroved the final version of the manuscript.

## Conflict of interest

The authors declare that the research was conducted in the absence of any commercial or financial relationships that could be construed as a potential conflict of interest.

## Publisher’s note

All claims expressed in this article are solely those of the authors and do not necessarily represent those of their affiliated organizations, or those of the publisher, the editors and the reviewers. Any product that may be evaluated in this article, or claim that may be made by its manufacturer, is not guaranteed or endorsed by the publisher.
